# Flavonoid Compound Icariin Activates Hypoxia Inducible Factor-1α in Chondrocytes and Promotes Articular Cartilage Repair

**DOI:** 10.1371/journal.pone.0148372

**Published:** 2016-02-03

**Authors:** Pengzhen Wang, Fengjie Zhang, Qiling He, Jianqi Wang, Hoi Ting Shiu, Yinglan Shu, Wing Pui Tsang, Shuang Liang, Kai Zhao, Chao Wan

**Affiliations:** 1 Ministry of Education Key Laboratory of Regenerative Medicine (Jinan University - The Chinese University of Hong Kong), Guangzhou 510000, China; 2 School of Biomedical Sciences Core Laboratory, Institute of Stem Cell, Genomics and Translational Research, Shenzhen Research Institute, The Chinese University of Hong Kong, Shenzhen 518057, China; 3 Ministry of Education Key Laboratory of Regenerative Medicine (The Chinese University of Hong Kong - Jinan University), School of Biomedical Sciences, Faculty of Medicine, The Chinese University of Hong Kong, Shatin, Hong Kong SAR, China; 4 Department of Microbiology, University of Alabama at Birmingham, Birmingham, AL 35294, United States of America; 5 Department of Chemistry, Faculty of Science, The Chinese University of Hong Kong, Shatin, Hong Kong SAR, China; INSERM - university Paris 7, FRANCE

## Abstract

Articular cartilage has poor capability for repair following trauma or degenerative pathology due to avascular property, low cell density and migratory ability. Discovery of novel therapeutic approaches for articular cartilage repair remains a significant clinical need. Hypoxia is a hallmark for cartilage development and pathology. Hypoxia inducible factor-1alpha (HIF-1α) has been identified as a key mediator for chondrocytes to response to fluctuations of oxygen availability during cartilage development or repair. This suggests that HIF-1α may serve as a target for modulating chondrocyte functions. In this study, using phenotypic cellular screen assays, we identify that Icariin, an active flavonoid component from Herba Epimedii, activates HIF-1α expression in chondrocytes. We performed systemic *in vitro* and *in vivo* analysis to determine the roles of Icariin in regulation of chondrogenesis. Our results show that Icariin significantly increases hypoxia responsive element luciferase reporter activity, which is accompanied by increased accumulation and nuclear translocation of HIF-1α in murine chondrocytes. The phenotype is associated with inhibiting PHD activity through interaction between Icariin and iron ions. The upregulation of *HIF-1α* mRNA levels in chondrocytes persists during chondrogenic differentiation for 7 and 14 days. Icariin (10^−6^ M) increases the proliferation of chondrocytes or chondroprogenitors examined by MTT, BrdU incorporation or colony formation assays. Icariin enhances chondrogenic marker expression in a micromass culture including *Sox9*, *collagen type 2* (*Col2α*1) and *aggrecan* as determined by real-time PCR and promotes extracellular matrix (ECM) synthesis indicated by Alcian blue staining. ELISA assays show dramatically increased production of aggrecan and hydroxyproline in Icariin-treated cultures at day 14 of chondrogenic differentiation as compared with the controls. Meanwhile, the expression of chondrocyte catabolic marker genes including *Mmp2*, *Mmp9*, *Mmp13*, *Adamts4* and *Adamts5* was downregulated following Icariin treatment for 14 days. In a differentiation assay using bone marrow mesenchymal stem cells (MSCs) carrying HIF-1α floxed allele, the promotive effect of Icariin on chondrogenic differentiation is largely decreased following Cre recombinase-mediated deletion of HIF-1α in MSCs as indicated by Alcian blue staining for proteoglycan synthesis. In an alginate hydrogel 3D culture system, Icariin increases Safranin O positive (SO+) cartilage area. This phenotype is accompanied by upregulation of HIF-1α, increased proliferating cell nuclear antigen positive (PCNA+) cell numbers, SOX9+ chondrogenic cell numbers, and Col2 expression in the newly formed cartilage. Coincide with the micromass culture, Icariin treatment upregulates mRNA levels of *Sox9*, *Col2α1*, *aggrecan* and *Col10α1* in the 3D cultures. We then generated alginate hydrogel 3D complexes incorporated with Icariin. The 3D complexes were transplanted in a mouse osteochondral defect model. ICRS II histological scoring at 6 and 12 weeks post-transplantation shows that 3D complexes incorporated with Icariin significantly enhance articular cartilage repair with higher scores particularly in selected parameters including SO+ cartilage area, subchondral bone and overall assessment than that of the controls. The results suggest that Icariin may inhibit PHD activity likely through competition for cellular iron ions and therefore it may serve as an HIF-1α activator to promote articular cartilage repair through regulating chondrocyte proliferation, differentiation and integration with subchondral bone formation.

## Introduction

Articular cartilage, a highly organized avascular connective tissue with substantial durability, has limited regenerative capacity following trauma or degenerative pathology. The deficit is likely due to insufficiency of resident mesenchymal stem cells (MSCs) and low metabolic activity of chondrocytes in articular cartilage. Current therapeutic strategies for articular cartilage repair have two main focuses: marrow stimulation and cell/tissue-based transplantation [1]. The marrow stimulating surgeries such as transcortical Pridie drilling, abrasion arthroplasty and microfracture [2,3] induce blood supply and recruit local stem/progenitor cells into the affected lesion from bone marrow through the subchondral bone. The cell/tissue-based transplantations fill the cartilage defects and promote regeneration with autologous chondrocytes, osteochondral allografts, cartilage allografts, or MSCs [4, 5]. Both strategies often include biomaterials as scaffolds combined with biomechanical or biochemical signals to better fill the defect areas, enhance marrow stimulation, maintain chondrogenic phenotype, or promote chondrogenesis *in vivo* or in *ex vivo* cultures [1]. The biochemical signals widely studied are growth factors such as TGF beta family members (e.g. TGF-β1, 2, 3, and BMPs), IGF-1 and FGF-2 that are identified as functional stimuli to promote chondrogenic differentiation and cartilage growth [6–11]. However, the exogenous growth factors are costly and subject to quick degradation, and their clinical efficacy and safety remain to be established, raising the demand for novel, effective, safe, bio-stable and low-cost alternatives.

Chondrogenesis is regulated by multiple mechanisms including the aforementioned growth factors emanating from the surrounding matrix, cytokines, oxygen supply and mechanical force, among which hypoxia is a hallmark for articular cartilage development and regeneration and functions as a stimulus for initiation of gene programs regulating chondrogenic cell proliferation, differentiation and metabolism. The MSCs or chondrocytes are readily located in a hypoxic microenvironment, and respond to the oxygen fluctuations through transcription factor hypoxia inducible factor-alpha (HIF-α) during cartilage development and repair. HIF-α is identified as a key mediator for oxygen sensing by mammalian cells. HIF is an αβ heterodimer, in which the α subunit is inducible by hypoxia, whereas the β subunit is constitutively expressed in the nucleus [12]. HIF-α exists as three isoforms HIF-1α, HIF-2α, and HIF-3α. Under normoxia, HIF-1α is hydroxylated by prolyl hydroxylase at specific proline residues, ubiquitinated through interaction with the von Hippel-Lindau tumor suppressor protein (pVHL), and subsequently degraded by the proteasome. Under hypoxia, the activity of the HIF-targeting prolyl hydroxylase enzymes (PHD1, PHD2, and PHD3) is inactivated, therefore HIF-1α is not hydroxylated and accumulated in the cytoplasm, and then translocates into the nucleus and dimerizes with HIF-1β to transactivate downstream target genes [13].

Using genetic approach, it is shown that HIF-1α is required for chondrocyte survival and growth arrest during development [14]. HIF-1α deletion in mouse limb bud mesenchyme leads to significantly reduced expression of Sox9, a key transcription factor of chondrocyte differentiation, in hypoxic prechondrogenic condensation resulting in differentiation arrest and thus severe skeletal malformations [15]. HIF-1α-null hypoxic chondrocytes exhibits significantly decreased aggrecan and Col2 expression, implying the important role of HIF-1α in controlling hypoxia-induced ECM synthesis in chondrocytes [16]. It is also demonstrated that the HIF-1α pathway is activated during bone regeneration [17–20]. Prolyl hydroxylase inhibitor, Deferoxamine (DFO), as an HIF-1α activator, improves skeletal repair through promoting angiogenesis and cartilaginous callus formation [20]. The HIF-1α downstream target erythropoietin (EPO) promotes bone healing through enhancing cartilaginous callus formation and angiogenesis [21]. Hypoxic conditions enhance cartilage formation of human MSCs and suppress their hypertrophic differentiation [20, 21]. The above information indicates that the hypoxia/HIF-1α pathway may serve as a molecular target to facilitate chondrogenesis.

In this study, based on cellular screen and functional assays, we identify Icariin, a flavonoid compound derived from Herba Epimedii, as an activator of HIF-1α in chondrocytes. Chondrocytes treated with Icariin have increased mRNA and protein levels and nuclear translocation of HIF-1α. Icariin increases the proliferation and enhances differentiation and ECM production of chondrocytes in micromass or 3D alginate hydrogel cultures, which is associated with upregulation of HIF-1α. In an osteochondral defect model in mice, Icariin promotes articular cartilage repair with improved ICRS II histological score compared with the controls. These results suggest that Icariin may serve as an HIF-1α activator to promote articular cartilage repair through regulating chondrocyte proliferation, differentiation and ECM synthesis.

## Materials and Methods

### Chemical and reagents

Icariin was obtained from Beijing Aoke Biotechnology CO. LTD (A0145). Stock solutions of icariin were prepared in dimethyl sulfoxide (DMSO) (Sigma Co, St Louis, Mo) and stored at −20°C. The final concentration of dimethyl sulfoxide used in the culture was 0.01% (v/v). Iron(II) sulfate heptahydrate (FeSO_4_) was purchased from Sigma (Sigma Co, St Louis, Mo).

### Luciferase reporter assay

7000 C2C12 cells were seeded in 96-well plates for 24 h and transfected with 100ng of HRE-Luc reporter construct using Lipofectamine LTX reagent. 0.05μg of pRL-CMV plasmid expressing Renillia luciferase was co-transfected in each well to monitor the transfection efficiency (Promega). C2C12 cells, a mouse myoblast cell line, had been used in HRE-Luc reporter assays [22]. At 24 h post-transfection, cells were maintained in normal medium with or without Icariin for further 24 h. Luciferase activity was measured by performing dual-luciferase reporter assay as described by the manufacturer (Promega). Relative luciferase activity was normalized with Renilla luciferase activity in each well and then compared the luciferase intensity of Icariin treated group with the control group.

### Isolation and culture of murine chondrocytes and bone marrow MSCs

Primary chondrocytes were isolated from the growth plates of newborn C57BL/6 mice using the method adapted from previously established protocol [23]. Briefly, the growth plates were digested by collagenase I and collagenase D, and chondrocytes were plated onto a 100 mm petri dish with DMEM (GIBCO, Invitrogen) supplemented with 1% (v/v) penicillin/streptomycin sulfate (GIBCO, Invitrogen), 1% (v/v) L-glutamine (GIBCO, Invitrogen) and 10% (v/v) fetal bovine serum (FBS, GIBCO, Invitrogen) at 37°C in a 5% CO_2_ humidified incubator under normoxic conditions (21% oxygen). On the second day, non-adherent cells were removed and the cultures were gently rinsed with PBS twice and cultured in the fresh medium. Adherent cells were cultured and medium was changed every 2 days until the cells became confluent. The chondrocytes were also cultured under hypoxic conditions (2% oxygen). Cells at early passages (no more than 3 passages) were used to perform the experiments so as to minimize the possibility of phenotypic changes over prolonged culture. Cells at the same passage were used within each experiment to ensure the comparability among groups. Bone marrow mesenchymal stem cells (MSCs) were isolated from 4-week old mice carrying HIF-1α floxed allele (HIF-1α^*flox/flox*^) by flushing out the bone marrow from the long bones using phosphate-buffered saline (PBS), followed by replating and adhesion culture of the bone marrow mononuclear cell population. MSCs colonies were expanded for 14 days and then passaged for sub-culture. MSCs were maintained in alpha-Minimum Essential Medium (α-MEM, GIBCO, Invitrogen) supplemented with penicillin (100 U/ml), streptomycin sulfate (100 g/ml), 1% (v/v) L-glutamine and 15% (v/v) fetal bovine serum (FBS) (GIBCO, Invitrogen) at 37°C in a 5% CO_2_ humidified incubator.

### UV-Vis spectrum assay

Stock solutions of FeSO_4_ and Icariin were prepared in deionized water (DI water) and DMSO, respectively. The stock solutions were further diluted with DI water to the desired concentration. All the UV-Vis spectra were recorded on a Cary 5G UV-VIS-NIR spectrophotometer at room temperature.

### MTT assay

The MTT method was used to evaluate the effect of icariin on chondrocytes proliferation. After 3 days of culture, the supernatant was removed, and 10 μl of MTT solution (5 mg/ml) was added to every well and incubated for 4 h before termination. After removal of the medium, the formed formazan pigment was dissolved with DMSO. The 150 μl of pigment solution was added to each well of a 96-well multi-plate and the absorbance was measured by using a microplate reader at 570 nm. All experiments were carried out in quadruplicate.

### Bromodeoxyuridine (BrdU) incorporation assay

Cell proliferation was examined by using the cell proliferation ELISA BrdU assay (Roche) according to the manufacturer’s instructions. Briefly, chondrocytes were cultured until confluency, then re-plated in 96-well plates at a density of 5 × 10^3^ cells in a final volume of 100 μl per well. The chondrocytes were treated with different concentrations of Icariin for 1 day or 3 days followed by BrdU (10 μM) treatment for 4 h. Then, the cells were fixed and incubated with peroxidase-conjugated anti-BrdU antibody for 90 min. BrdU incorporation was detected by incubating the cells with tetramethyl-benzidine (TMB) as a substrate. Color development, which was directly proportional to the amount of DNA synthesis and hereby to the number of proliferating cells, was quantified by measuring the absorbance at 370 nm by a microplate reader. The experiments were carried out in triplicate.

### Colony formation assay

The primary chondrogenic cells isolated from growth plates of newborn mice were treated with different concentrations of Icariin for 24 h, then trypsinized and replated. 500 cells were plated in 6-well culture plates in a CO_2_ incubator supplied with 5% CO_2_ for 2 weeks. Cells were fixed in 10% (v/v) formalin for 10 min and stained with 0.1% (w/v) crystal violet (Sigma) for 10 min. The colonies (>50 cells) were counted using a dissecting microscope. The single cells that form colonies containing more than 50 cells were referred to chondroprogenitors. The experiments were performed in triplicate.

### Micromass culture and Alcian blue staining

Chondrocytes were seeded in a 4-well plate at a density of 1.0 × 10^5^ cells per well in a final volume of 10 μl and incubated for 4 h at 37°C to allow adherence. 0.5 ml of fresh media was added to each well. On the second day, media were changed to chondrogenic medium containing various concentrations of Icariin. The chondrogenic medium contained Dulbecco’s modified Eagle’s medium (DMEM) (high glucose) with 10% (v/v) fetal bovine serum (FBS), ITS+ Premix 1:100 dilution, 1% (v/v) penicillin/streptomycin, 100 mM L-glutamine, 50 μg/ml ascorbic acid (pH 7.1) and 10 mM -glycerophosphate (Sigma). After 7 or 14 days, wells were washed once with PBS and then fixed for 20 min with 0.5 ml of 10% (v/v) neutral buffered formalin. The pellets were then washed three times with sterile distilled, deionized water, with the last wash being left on the cells for 15 min. Subsequently, 0.5 ml of 1% (w/v) Alcian blue (Sigma) in glacial acetic acid (3%) (v/v) was added to each well for 30 min or 1 h at room temperature. After two washes with 70% (v/v) ethanol and three washes with sterile distilled, deionized water, photomicrographs of the stained cell mass were obtained. The ECM proteoglycan was stained blue.

### ELISA assay

Hydroxyproline and aggrecan in chondrocytes were assayed using ELISA kits according to manufacturer’s recommendations. ELISA kits used were mouse hydroxyproline ELISA kit (CUSABIO) and mouse AGC ELISA kit (HXBIO). Optical density was determined using a microplate reader. All experiments were carried out in triplicate.

### Three-dimensional (3D) alginate-chondrocytes beads culture

Chondrocytes were trypsinized and resuspended in 2% (w/v) alginate solution in 0.9% (w/v) sodium chloride at a concentration of 4 × 10^6^ cells/ml and mixed well by pipetting up and down. The alginate/cell mixture (20 μl) was dropped into sterile 102 mM Calcium Chloride solution. The 3D alginate-chondrocytes beads were formed in the Calcium Chloride solution for three minutes and then washed twice with 0.9% (w/v) Sodium Chloride. The 3D alginate-chondrocytes beads were then transferred to a culture dish containing chondrogenic medium with or without Icariin (10^−6^ M) and cultured in the incubator at 37°C. The medium was changed every 3 days up to 21 days. The alginate/cell complexes were processed for mRNA expression, histological characterization and immunohistochemistry analysis.

### Osteochondral defect model in mice

Experimental procedures were carried out according to protocols approved by Animal Experimentation Ethics Committee, The Chinese University of Hong Kong and Animal (Control of Experiments) Ordinance from Department of Health, Hong Kong SAR. The osteochondral defect model in mice was adapted from previously established procedure [24]. Briefly, the C57BL/6 male mice (20–25 g) were anesthetized using xylazine (5 mg/kg) and Ketamine (40 mg/kg) cocktail, under sterile conditions. The inter-chondyle notch of the distal femur was exposed and an osteochondral defect with 1 mm in diameter and 2 mm in height was created with a 21 G needle. The defects were implanted with the 3D alginate-Gelfoam complexes incorporated with or without Icariin. Before the surgery, the 3D alginate-Gelfoam complexes were generated as follows. Icariin was dissolved in 4% (w/v) alginate acid sodium (Sigma) solution to make a working solution at 1 x 10^−6^ M. All the solutions were freshly prepared. The Gelfoam^®^ was then pre-humidified with sterile deionized, distilled H_2_O and dried in sterilized gauze, and was shaped in designed cubic structure (2 X 2 X 2 mm^3^). 10 μl of alginate solution containing Icariin or no Icariin was carefully added into the 3D Gelfoam scaffold followed by immersing with 102 mM calcium chloride solution. The implants were then incubated in 37°C for 5 minutes. The 3D complexes were trimmed to 1 mm in diameter and 2 mm in height for implantation. 5 mice were used in each group. After surgery, the animals were administered with analgesics (Buprenorphine HCl, 0.02 mg/Kg/day) once per day for three days. The animals were monitored every day post-surgery in the first week, then two times each week up to 12 weeks. There is no unintended death of animal during the study. Sample harvesting and autopsy examination were performed at 2 weeks, 6 weeks and 12 weeks post-surgery, the animals were sacrificed by cervical dislocation under anaesthesia with 2% (v/v) Isoflurane. The cartilage samples of the distal femur were processed for histological analysis.

### Histology and immunohistochemistry

For histologic evaluation, the samples were fixed in 4% (w/v) paraformaldehyde (PFA) in PBS, decalcified, and embedded with paraffin. The sections were cut with a microtome at the thickness of 5 μm. The sections were stained with Hematoxylin & Eosin or SO/fast green staining. For the 3D culture system, alginate/chondrocytes complexes were rinsed in PBS at 4°C, fixed for 24 h with 4% (w/v) PFA, soaked in a series of gradient sucrose, embedded in OCT, and solidified by liquid nitrogen. Frozen sections were cut for standard Hematoxylin & Eosin, Alcian blue, and SO/fast green staining. For immunohistochemistry analysis, endogenous peroxidase activity was quenched by 3% (v/v) hydrogen peroxide. The sections were blocked with 3% (v/v) bovine serum albumin (BSA) and incubated with the primary antibodies of rabbit polyclonal anti-Sox9 (Abcam), rabbit polyclonal anti-Collagen type II (Col2), rabbit polyclonal anti-HIF-1α (Novus Biologicals) and mouse monoclonal anti-PCNA (proliferating cell nuclear antigen) (Cell Signaling) respectively. Adjacent sections were incubated with IgG as negative controls. Histostain Plus Kit was used for color development with diaminobenzidine (DAB) according to the manufacturer’s instructions (Invitrogen).

### Histological assessment

The International Cartilage Repair Society (ICRS) II score was used to assess the *in vivo* cartilage repair [25]. The central portion of the osteochondral defect was defined as region of interest and serial adjacent sections were employed for analysis. Three blinded readers graded the cartilage sections according to ICRS II parameters and criteria. The adapted histological parameters include: (1) matrix staining (metachromasia): 0%, no staining; 100%, full metachromasia; (2) subchondral bone: 0%, abnormal; 100%, normal subcondral bone; (3) overall assessment: 0%, bad (fibrous tissue); 100%, good (hyaline cartilage).

### Immunoblotting

Western blotting analysis was performed using standard protocol. In brief, whole-cell lysate was obtained by using cell lysis buffer in the presence of a protease inhibitor cocktail (Sigma). Equal amount of protein was loaded onto an SDS mini-PAGE system after concentrations were determined, and transferred onto a PVDF membrane using a Bio-Rad semi-dry transfer system. Protein transfer efficiency and size determination were verified using prestained protein markers. Membranes were blocked with 5% (w/v) dry milk in Tris-buffered saline with Tween-20 for 1 h at room temperature and subsequently incubated overnight with primary antibodies at 4°C. The primary antibodies used included anti-HIF-1α, anti-EGLN2/PHD1, anti-EGLN3/PHD3 (Novus Biologicals, Littleton, CO) and anti-PHD2 (H-40) (Santa Cruz, CA). Signals were detected using an HRP-conjugated secondary antibody and the SuperSignal West Femto Maximum Sensitivity Substrate (Pierce, Thermo Fisher Scientific, Inc., Rockford, IL, USA). The quantitation of immunoblotting bands was performed using NIH Image J 1.48 software. The values were obtained after normalization using the beta-actin signal intensity and compared with that of the non-treatment control.

### Immunofluorescence and confocal microscopy

Chondrocytes were plated on lysine-treated glass coverslips placed in 6-well plates. After culturing for 24 h under normal condition, cells were starved for 12 h and treated with Icariin (10^−6^ M) for 8 h. The cells were then fixed with 4% (w/v) PFA for 10 min at room temperature, permeabilized with 0.1% (v/v) Triton X-100 for 3 minutes, and incubated with the primary antibody of rabbit anti-HIF-1α (Novus, 1:100) overnight at 4°C. After rinsing, the coverslips were incubated with fluorescence conjugated secondary antibody (Molecular Probes; Invitrogen) and protected from light for 30 min at room temperature, and finally mounted in VECTASHIELD mounting media with DAPI (Vector Laboratories). Fluorescence localization was detected by confocal microscope with a laser-scanning microscope (FV10-ASW 1.7; Olympus).

### Quantitative real-time polymerase chain reaction (qPCR)

Total RNA was extracted from chondrocytes using TRIzol reagent (Invitrogen). First strand cDNA was synthesized from 1 μg of total RNA in the presence of oligo-dT12-18 primer (Invitrogen) and MMLV reverse transcriptase according to manufacturer’s instructions (Promega). Quantitative real-time PCR was performed with SYBR Premix ExTaq (Takara) in ABI Fast Real-time PCR 7900HT System (Applied Biosystems). All samples were performed in triplicate. β-actin was used as an internal control. Primer sequences were shown in Table 1.

**Table 1:** Sequences of Primers Used for Quantitative Real-time PCR.

Gene	Forward primer	Reverse primer
Sox9	AGGAAGCTGGCAGACCAGTA	TCCACGAAGGGTCTCTTCTC
Col2α1	CTACGGTGTCAGGGCCAG	GCAAGATGAGGGCTTCCATA
Aggrecan	CTGAAGTTCTTGGAGGAGCG	CGCTCAGTGAGTTGTCATGG
HIF-1α	TGGCTCCCTATATCCCAATG	GGTCTGCTGGAACCCAGTAA
Clo10α1	CCTGGTTCATGGGATGTTTT	ACCAGGAATGCCTTGTTCTC
Mmp2	CCAGCAAGTAGATGCTGCCT	GATGGCATTCCAGGAGTCTG
Mmp9	TAGCTACCTCGAGGGCTTCC	GTGGGACACATAGTGGGAGG
Mmp13	CTGGACCAAACTATGGTGGG	GGTCCTTGGAGTGATCCAGA
Adamts4	CAGTGCCCGATTCATCACT	GAGTCAGGACCGAAGGTCAG
Adamts5	CGAAGAGCACTACGATGCAG	TGGAGGCCATCATCTTCAAT
β-actin	GTTGTCGACGACGAGCG	GCACAGAGCCTCGCCTT

### Statistical analysis

All data were expressed as Mean ± SD and analyzed using SPSS version 17.0 software (SPSS Inc., Chicago, IL). The normality of the data and the equal variances across the groups were validated by the Shapiro-Wilk’s test and the Levene’s test respectively. The differences between groups were analyzed by using Student’s t-test or ANOVA. All experiments were performed in triplicate unless otherwise stated. *P* < 0.05 was defined as significance level.

## Results

### Icariin activates HIF-1α expression in chondrocytes by inhibiting PHD through competition for iron ions

**Fig 1:**
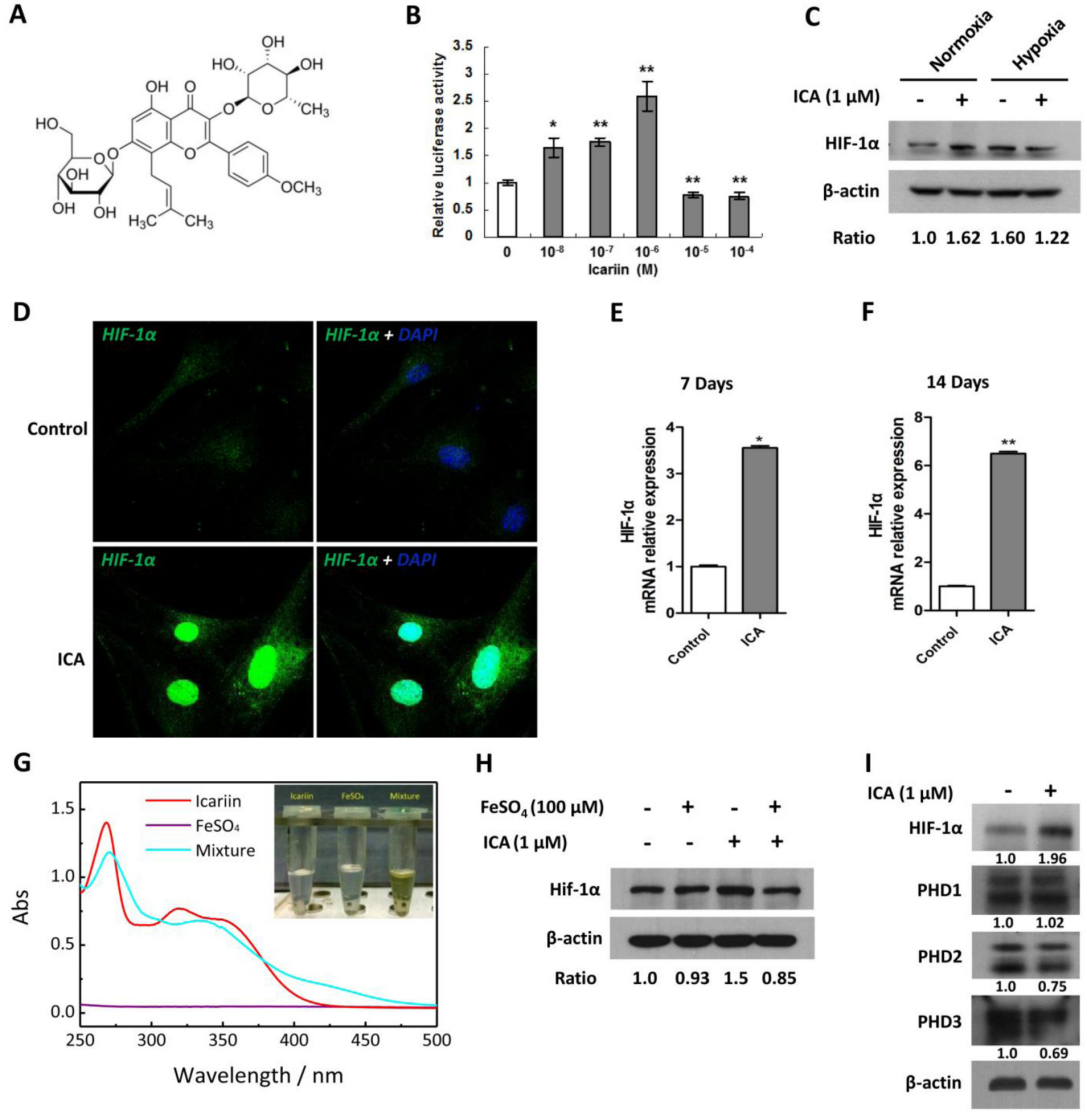
Icariin upregulates HIF-1α expression in chondrocytes by inhibiting PHDs activity through competition for iron ions. (A) The chemical formula of Icariin. (B) Hypoxia response element luciferase reporter assay in C2C12 cells treated with Icariin at indicated concentrations. (C) Western blot analysis for HIF-1α protein expression in primary culture-derived chondrocytes under normoxia or hypoxia or treated with or without Icariin (10^−6^ M) for 8 h. β-actin used as the loading control. (D) Detection of HIF-1α nuclear localization in Icariin (10^−6^ M)-treated chondrocytes by immunofluorescence staining under confocal microscope. (E, F) Chondrocytes were cultured and induced to differentiate in chondrogenic medium in the presence or absence of Icariin (10^−6^ M) for 7 or 14 days. HIF-1α mRNA levels were detected by real-time PCR in Icariin-treated chondrocytes compared with that of the control cells. *P < 0.05, **P < 0.01, n = 3. (G) UV-Vis spectra of the Icariin, FeSO_4_ and their mixture (*n*_Icariin_: *n*_FeSO4_ = 3: 1, *C*_Icariin_ = 0.5mM) in aqueous solution after incubation at 37°C for 12 h; *C*_Icariin_ = 0.5mM; *C*_FeSO4_ = 1mM; The inset shows the visual appearance of each species. (H) Western blot analysis for HIF-1α protein expression in chondrocytes treated with or without Icariin (10^−6^ M) and FeSO_4_ (100 μM) for 12 h. (I) Western blot analysis for PHDs and HIF-1α protein expression in chondrocytes treated with or without Icariin (10^−6^ M) for 12 h. In all Figs, ICA, Icariin.

Icariin has a chemical formula C_33_H_40_O_15_ (Fig 1A) with molecular mass 676.662 g/mol. We screened Icariin for its ability to activate a hypoxia responsive element (HRE) construct driving a luciferase reporter (HRE-Luc) in C2C12 cells. Under normoxia (21% oxygen), Icariin increased luciferase reporter activity in C2C12 cells in a dose-dependent manner at concentrations ranging from 10^−8^ M to 10^−6^ M, while showed an inhibitory effect (Fig 1B) at higher doses (10^−5^ M and 10^−4^ M). The changes in HRE reporter activity in C2C12 cells upon Icariin treatment were in line with the upregulation of HIF-1α protein level (Fig 1C) and increased HIF-1α nuclear localization (Fig 1D) in primary culture-derived chondrocytes following treatment with Icariin for 8 hours. Icariin stimulated an increase in HIF-1α protein level in the chondrocytes under normoxia by 1.62 fold, which was comparable to the 1.60-fold accumulation of HIF-1α when the chondrocytes were exposed to hypoxia (2% oxygen), while no additive or synergistic effect was observed when the cells were treated with Icariin under hypoxia (Fig 1C). When the chondrocytes were subjected to chondrogenic differentiation, Icariin significantly elevated *HIF-1α* mRNA levels following 7 or 14 days of treatment (Fig 1E and 1F). It was documented that some flavonoids (e.g. quercetin, galangin) chelated cellular iron ions, a required cofactor for PHD activity, leading to the inhibition of PHDs-catalyzed HIF prolyl hydroxylation and subsequently HIF-1α/2α accumulation [26]. We thus determined whether Icariin, as a flavonoid component, induced the accumulation of HIF-1α in chondrocytes through the similar mechanism as other flavonoids [27]. We employed UV-Vis spectrum assays to compare the difference in the absorption spectra of Icariin, FeSO_4_ and their mixture solutions. After adding FeSO_4_ to Icariin solution and being incubated at 37°C for 12 h, there was a clear red shift of the absorption peaks originally located at *λ* = 268nm and *λ* = 319nm for Icarrin solution. Moreover, a new absorption domain in the visible light region at the *λ* = 425nm was observed in the mixture solution, which did not exist either in Icariin or FeSO_4_ solution (Fig 1G). This absorption domain has been predicted by the Density functional theory (DFT) computation using Gaussian03 in a system composed of ferrous ion and quercetin, as which Icariin has the same core structure [27]. The new absorption domain endows the light yellow color to the mixture, as shown in the inset of Fig 1G, while both Icariin and FeSO_4_ solutions were almost colorless at the same experimental condition, indicating that some new species formed after the mixing and incubation, probably caused by the interaction between Fe^2+^ and Icariin. Coincide with this observation, we found that the positive effect of Icariin on HIF-1α was eliminated by FeSO_4_ (Fig 1H), suggesting that Icariin might function as a HIF-1α activator by inhibiting PHD activity through competition for cellular iron ions. Interestingly, the protein expression of PHD2 and PHD3 but not PHD1 was also inhibited by Icariin treatment, which was in line with the upregulation of HIF-1α (Fig 1I).

### Icariin increases chondrocytes proliferation

**Fig 2:**
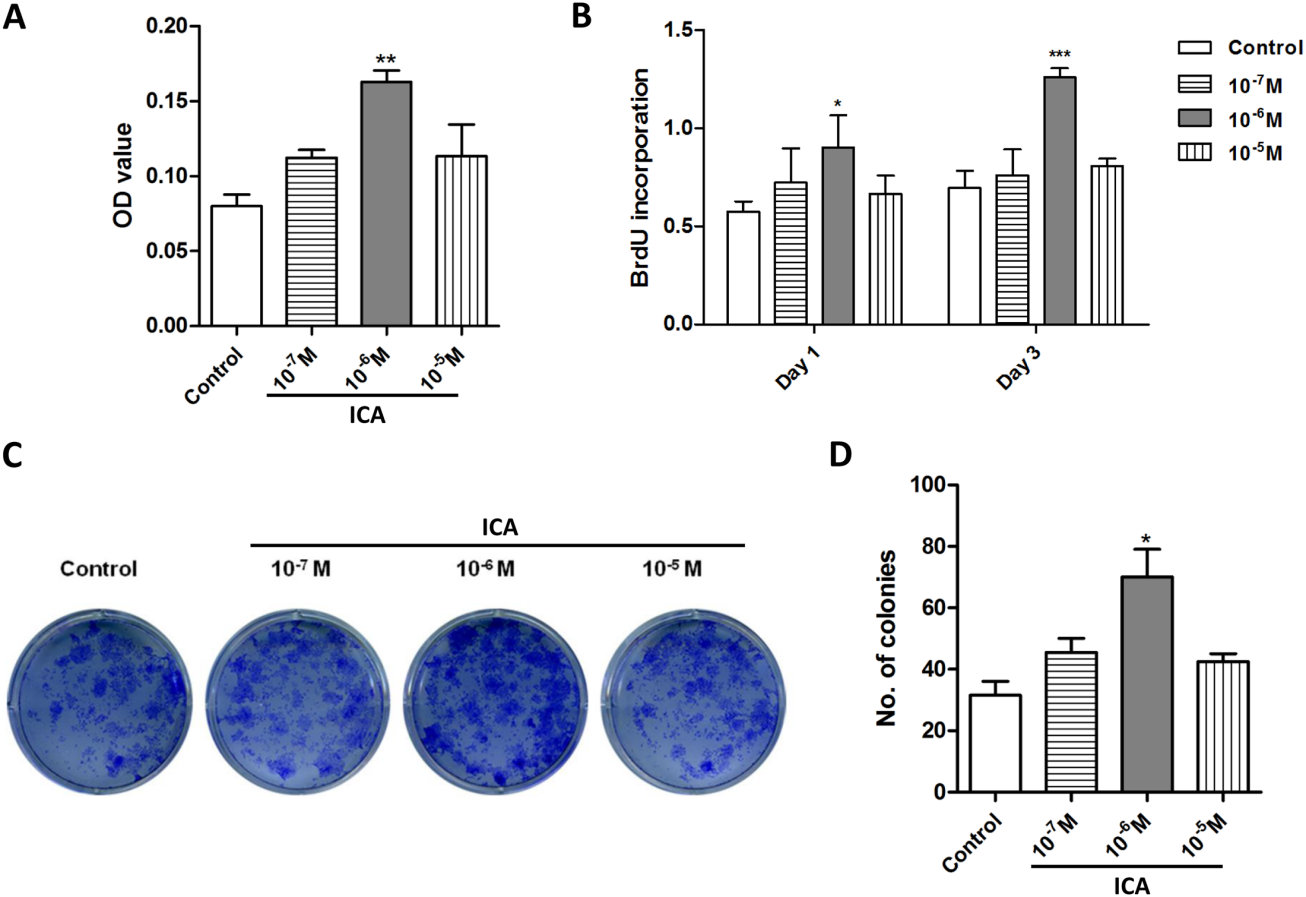
Icariin increases chondrocytes proliferation. (A) MTT assay for cell viability of chondrocytes treated with or without Icariin (0 M, 10^−7^ M, 10^−6^ M, 10^−5^ M) for 3 days. Treated groups compared with control group, *P < 0.05, **P < 0.01, n = 3. (B) BrdU incorporation assay for chondrocytes treated with or without Icariin (0 M, 10^−7^ M, 10^−6^ M, 10^−5^ M) for 1 day or 3 days. Treated groups compared with control group, *P < 0.05; ***P < 0.001, n = 3. (C) Colony formation assay for chondroprogenitor cells treated with Icariin (10^−7^ M, 10^−6^ M, 10^−5^ M) for 24 h followed by 14 days sub-culture. (D) Quantitation of the colony numbers from (C), *P < 0.05, n = 3.

We assessed the effect of Icariin on the viability of chondrocytes by MTT assay. Chondrocytes were treated with different concentrations of Icariin for 3 days. The results showed that the cells did not only survive the prolonged treatment of Icariin at a wide range of doses (from 10^−7^ M to 10^−5^ M) but also significantly increased their numbers and achieved the highest viability upon 10^−6^ M of Icariin treatment compared with the control group (Fig 2A). To further confirm that Icariin has a positive effect on proliferation of chondrocytes, we performed BrdU incorporation assay and colony-formation assay. As shown in Fig 2B, 10^−6^ M of Icariin increased the BrdU incorporation in chondrocytes to the level that was significantly higher than that in the control group after one day of culture and this effect was further enhanced after three days of culture. Colony formation assay showed that the chondroprogenitors treated with 10^−6^ M of Icariin for 24 h formed colonies by more than 2 folds compared with the group without treatment at the end of 2 weeks culture (Fig 2C and 2D). These data suggest that Icariin is a safe compound for the growth of chondrocytes and 10^−6^ M is an optimal dose to promote chondrocyte proliferation.

### Icariin enhances chondrogenic marker expression and cartilaginous matrix protein synthesis while down-regulates catabolic gene expression

**Fig 3:**
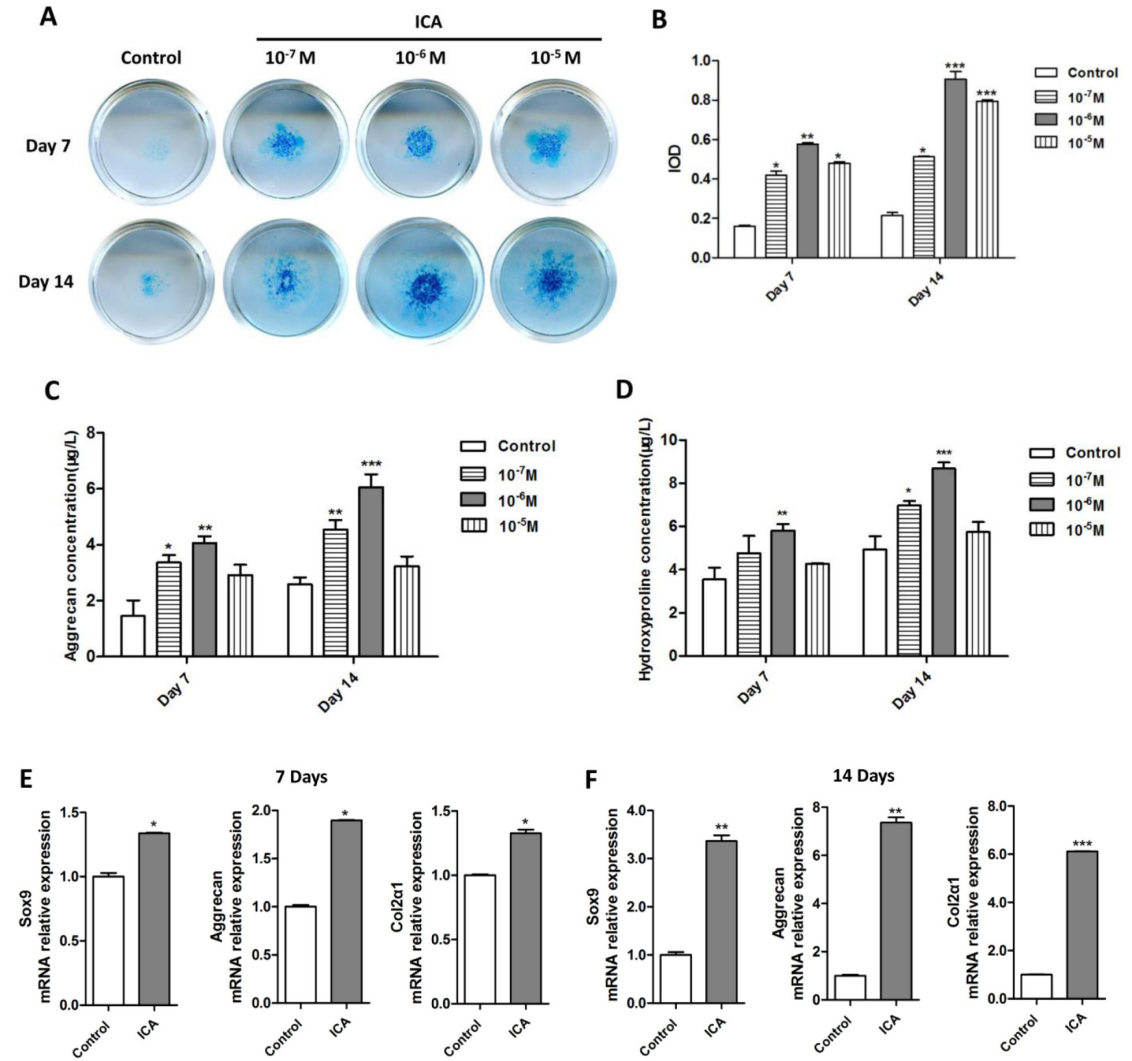
Icariin enhances chondrogenic marker expression and cartilage matrix synthesis while the effect is limited by knockdown of HIF-1α. (A) Chondrocytes were processed for micromass culture and induced to differentiate in chondrogenic medium in the presence or absence of Icariin (10^−7^ M, 10^−6^ M, 10^−5^ M). The cell masses were stained with Alcian blue after 7 or 14 days culture, respectively. Note that Icariin (10^−6^ M) increased proteoglycan synthesis. (B) Quantitation of the value of integral optical density (IOD) from (A). Treated groups compared with control group, *P < 0.05; **P < 0.01; ***P < 0.01, n = 3. (C, D) ELISA assays for production of aggrecan (C) and hydroxypoline (D) in chondrocytes. Icariin treated groups versus control groups, *P < 0.05; **P < 0.01; ***P < 0.001, n = 3. (E, F) Chondrocytes were cultured and induced to differentiate in chondrogenic medium in the presence or absence of Icariin (10^−6^ M) for 7 (E) or 14 (F) days. *Sox9*, *Col2α1* and *Aggrecan* mRNA expression was detected by real-time PCR in Icariin treated chondrocytes and compared with that in the control cells. *P < 0.05, **P < 0.01; ***P < 0.001, n = 3.

**Fig 4:**
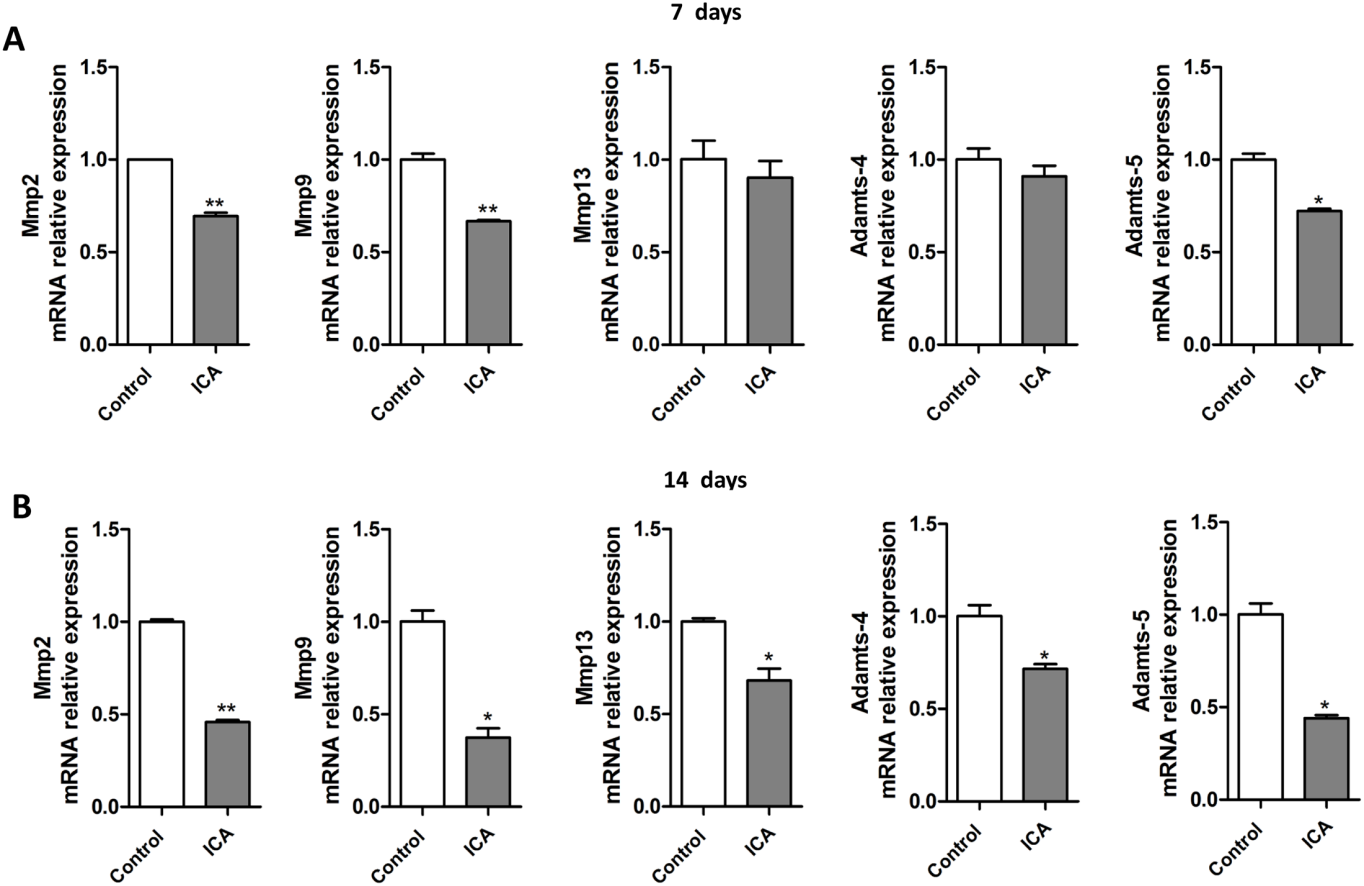
Icariin inhibits catabolic marker genes expression in chondrocytes. Chondrocytes were cultured and induced to differentiate in chondrogenic medium in the presence or absence of Icariin (10^−6^ M) for 7 (A) or 14 (B) days. *Mmp2*, *Mmp9*, *Mmp13*, *Adamts4* and *Adamts5* mRNA expression was detected by real-time PCR in Icariin treated chondrocytes compared with that of the control cells. *P < 0.05; **P < 0.01; n = 3.

We next examined the effect of Icariin on chondrocytes differentiation. Using chondrocyte micromass culture, we observed a persistent and marked increase in proteoglycan synthesis in the culture following treatment with different concentrations of Icariin (10^−7^ M to 10^−5^ M) at day 7 and day 14, as evident by the quantitative evaluation of positive Alcian blue staining (Fig 3A) using the integral optical density measurement (Fig 3B). We then performed the ELISA assays to specifically analyze the production of aggrecan, the major proteoglycan in the articular cartilage, as well as the hydroxyproline, an important component of collagen in cartilage tissue, in the chondrocyte micromass culture. Both aggrecan and hydroxyproline markedly increased after 10^−7^ M and 10^−6^ M of Icariin treatment at day 7 and/or day 14 (Fig 3C and 3D). Icariin at the concentration of 10^−6^ M showed the most dramatic effect on the production of proteoglycan, aggrecan and hydroxyproline (Fig 3B, 3C and 3D). The enhanced anabolic responses (synthesis and accumulation of cartilaginous matrix proteins) in the chondrocyte culture receiving 10^−6^ M of Icariin were accompanied by elevated expression levels of *Sox9*, *Col2α1* and *aggrecan* mRNA at day 7 (Fig 3E and 3F). The expression of these chondrogenic marker genes further elevated following Icariin treatment for 14 days (Fig 3F). The catabolism is another key metabolic process in chondrocytes, which involves MMP-mediated breakdown of cartilaginous tissues. It works with anabolism together to regulate cartilage matrix homeostasis. We then detected the effect of Icariin on the expression of key catabolic genes including *Mmp2*, *Mmp9*, *Mmp13*, *Adamts4* and *Adamts5*, among which *Mmp2*, *Mmp9* and *Adamts5* mRNA levels were downregulated at day 7 following Icariin treatment compared with that of the control (Fig 4A). The mRNA expression of all the tested catabolic genes was significantly downregulated following 14 days treatment with Icariin (Fig 4B). The results suggest that Icariin exerts positive effect on anabolic function but inhibits catabolic metabolism of chondrocytes, through which it may promote chondrocyte differentiation and regulate cartilage formation.

### The positive effects of Icariin on chondrocyte functions are partially mediated by HIF-1α

**Fig 5:**
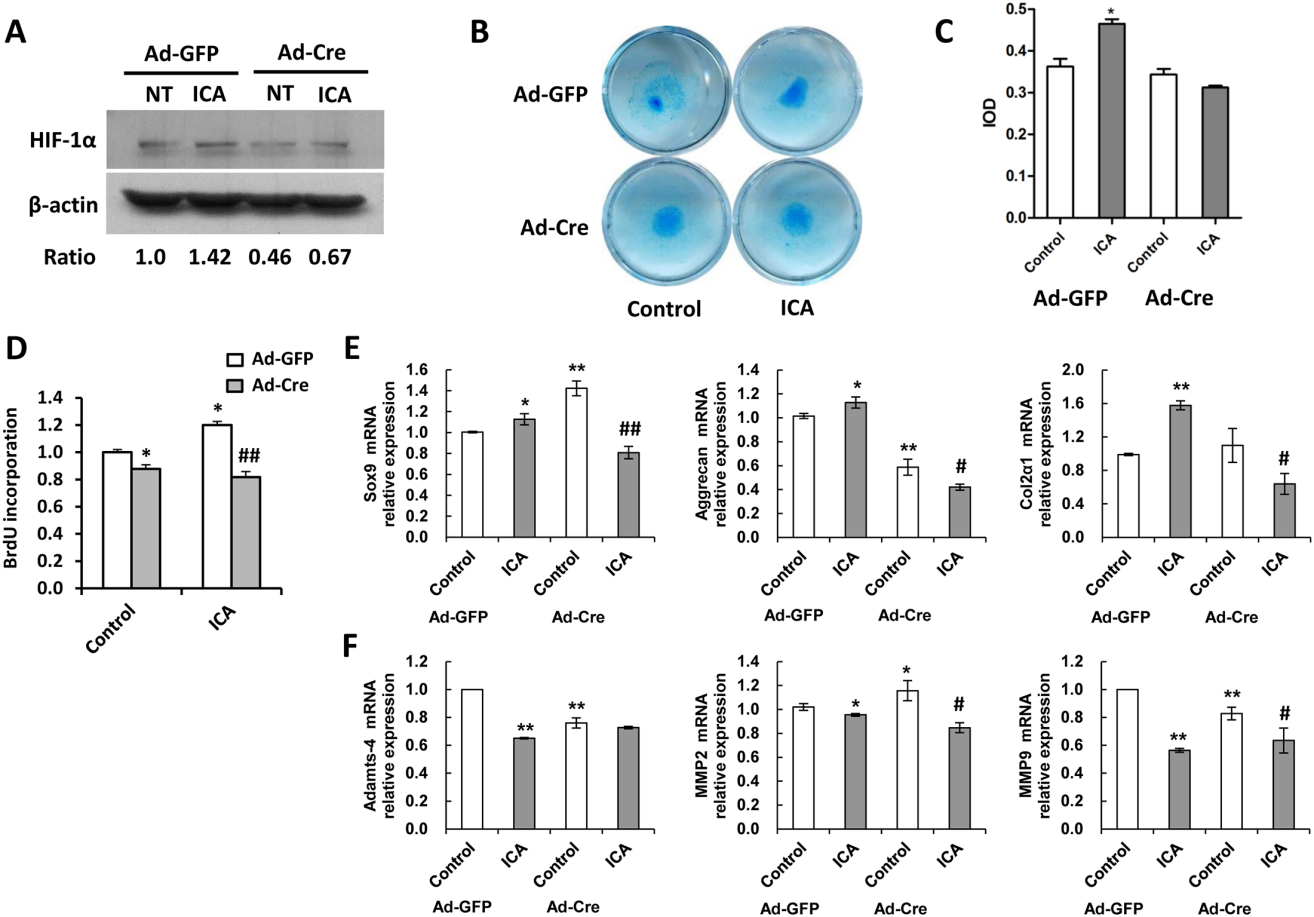
Deletion of HIF-1α eliminates the positive effects of Icariin on chondrocytes. (A) Western blot analysis for HIF-1α protein expression in MSCs (HIF-1α Floxed) following treatment with Ad-GFP or Ad-Cre and treated with or without Icariin (10^−6^ M) for 12 h. β-actin used as the loading control. (B) Alcian blue staining for preoteoglycan synthesis in MSCs (HIF-1α floxed) following treatment with Ad-GFP or Ad-Cre and treated with or without Icariin (10^−6^ M) for 14 days. (C) Quantitation of the value of integral optical density (IOD) from (B). Compared with Ad-GFP-treated control group, *P < 0.05; n = 3. (D) BrdU incorporation assay for chondrocytes following treatment with Ad-GFP or Ad-Cre and treated with or without Icariin (10^−6^ M) for 48 h. Compared with Ad-GFP-treated control group, *P < 0.05, **P < 0.01; n = 3. Compared with Ad-GFP-treated ICA control group, ^##^*P* < 0.01; n = 3. (E, F) Chondrocytes following treatment with Ad-GFP or Ad-Cre were cultured under normal medium in the presence or absence of Icariin (10^−6^ M). (E) *Sox9*, *Aggrecan* and *Col2α1* mRNA expression in chondrocytes was detected by real-time PCR. (F) *Adamts4*, *Mmp2*, and *Mmp9* mRNA expression in chondrocytes was detected by real-time PCR. Compared with Ad-GFP-treated control group,*P < 0.05, **P < 0.01; n = 3. Compared with Ad-Cre-treated control group, ^#^*P* < 0.05; ^##^*P* < 0.01; n = 3.

To determine whether the observed effects of Icariin on chondrocyte functions are HIF-1α dependent, parallel assays were performed in MSCs or chondrocytes carrying HIF-1α floxed allele, in which HIF-1α could be deleted by the Cre-loxp strategy. Western blot showed a more than 50% deletion of HIF-1α by adenovirus containing Cre recombinase (Ad-Cre) compared with the control (Ad-GFP) (Fig 5A). The promotive effect of Icariin on proteoglycan synthesis was largely reduced following Cre-mediated deletion of HIF-1α in MSCs as indicated by Alcian blue staining and its quantitation (Fig 5B and 5C). BrdU incorporation assay showed that the promotive effect of Icariin on chondrocyte proliferation was eliminated when HIF-1α was deleted (Fig 5D). Interestingly, real time-PCR analysis showed that the Icariin-elevated expression of chondrocyte anabolic marker genes *Sox9*, *Aggrecan* and *Col2α1* was not seen following deletion of HIF-1α (Fig 5E). Instead, Icariin significantly reduced the mRNA of these chondrocyte anabolic marker genes upon HIF-1α deletion, indicating that Icariin regulates the chondrocyte anabolic marker genes expression in a HIF-1α dependent manner. Deletion of HIF-1α also revealed a complicated interaction between HIF-1α and Icariin in control of chondrocyte catabolic marker genes expression (Fig 5F). The decreased mRNA expression of *Adamts4*, *MMP2* and *MMP9* upon Icariin treatment was eliminated, further enhanced and reduced respectively by HIF-1α deletion. Taken together, these data suggest that Icariin enhances chondrogenic proliferation, differentiation and cartilage matrix synthesis at least partially mediated by HIF-1α.

### Icariin enhances chondrogenesis in alginate-chondrocytes 3D culture system in association with upregulation of HIF-1α

**Fig 6:**
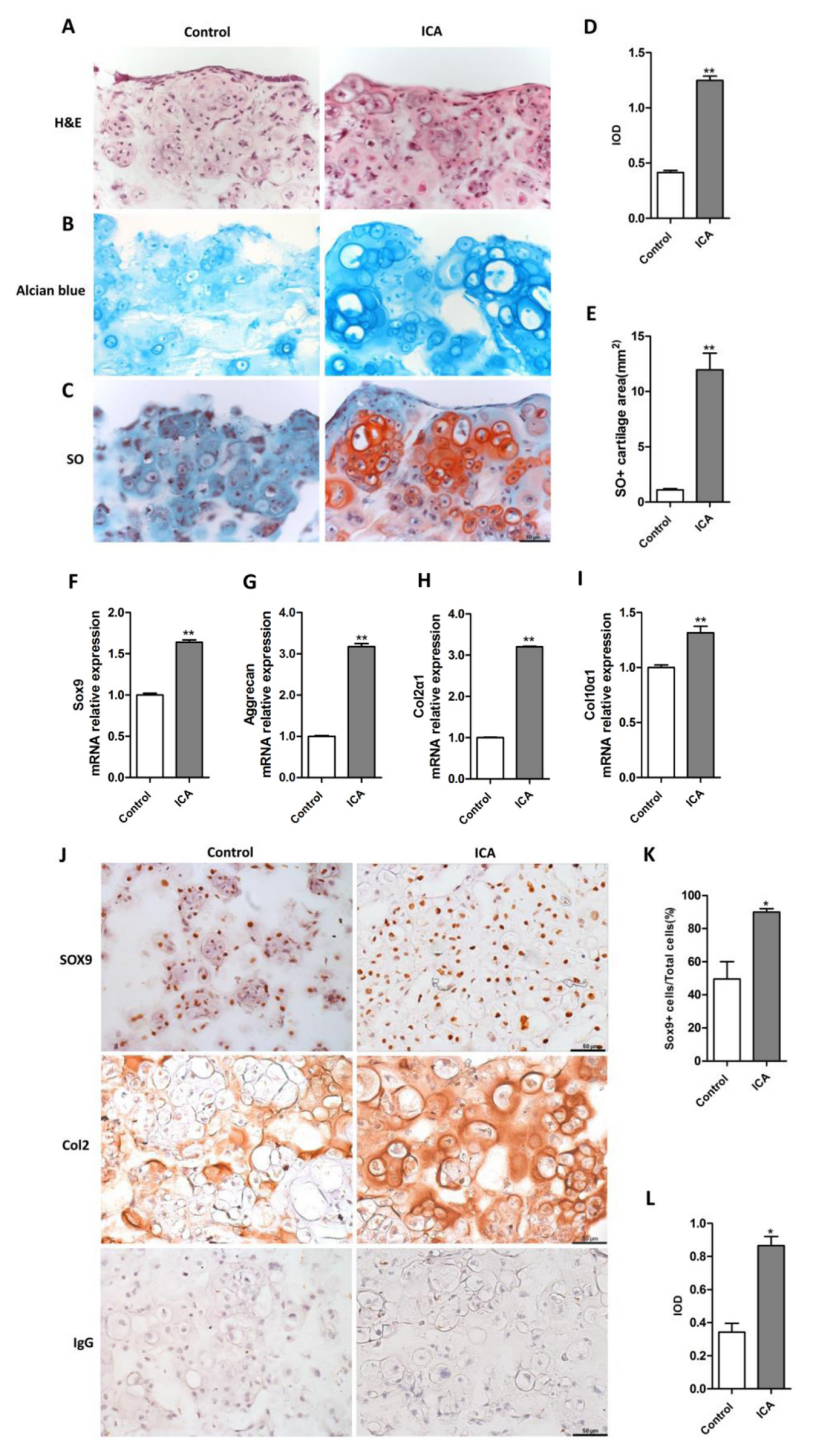
Icariin promotes chondrogenesis in alginate-chondrocyte 3D culture system. (A-C) Representative H&E, Alcian blue and SO histological images for sections from alginate-chondrocyte 3D culture system treated with Icariin (10^−6^ M) for 21 days, with none treatment as control. Scale bar = 50 μm. (D) Quantitation of IOD for Alcian blue staining. Icariin treated group compared with control group, **P < 0.01, n = 3. (E) Quantitation of the positive SO staining area. Icariin treated group compared with control group, **P < 0.01, n = 3. (F-I) The alginate-chondrocyte 3D cultures were treated with Icariin (10^−6^ M) for 21 days. *Sox 9*, *Aggrecan*, *Col2α1* and *Col10α1* mRNA expression was quantified by real-time PCR and compared with that of control group with no Icariin treatment. **P < 0.01, n = 3. (J) Representative images of the immunostaining for SOX9 and Col2 in the sections. IgG was used as negative control. Scale bar = 50 μm. (K) Quantitation of SOX9^+^ chondrocytes was presented as percentage of total chondrocytes in the SOX9 stained sections from (J). *P < 0.05, n = 3. (L) Densitometric analysis of Col2 immunostaining in (J) using GraphPad Prism 5 software. *P < 0.05, n = 3.

**Fig 7:**
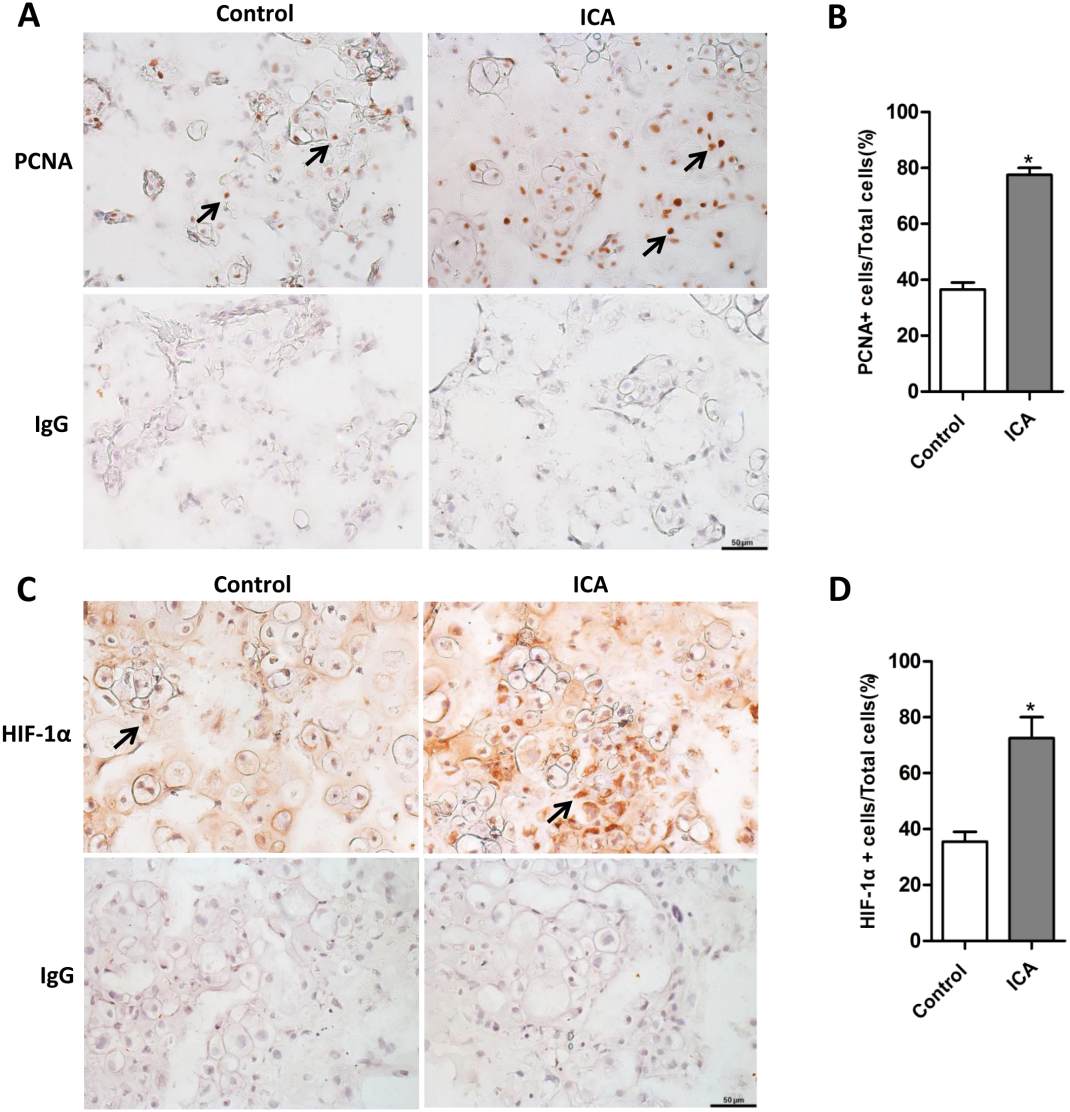
Icariin increases chondrocyte proliferation accompanied by upregulation of HIF-1α in alginate-chondrocyte 3D culture system. (A) Representative images of immunostaining for PCNA in 3D cultured sections from Icariin treated group and control group. Arrows indicate PCNA^+^ chondrocytes. IgG was used as negative control. Scale bar = 50 μm. (B) Quantitation of the percentage of PCNA positive cells in the Icariin treated group and control group. *P < 0.05, n = 3. (C) Representative images of immunostaining for HIF-1α in 3D cultured sections from Icariin treated group and control group. Arrows indicate HIF-1α positive (HIF-1α+) chondrocytes. IgG was used as negative control. Scale bar = 50 μm. (D) Quantitation of the percentage of HIF-1α+ cells in the sections from Icariin treated groups and control groups. *P < 0.05, n = 3.

To further define the effect of Icariin on chondrogenesis we employed an alginate-chondrocytes 3D culture system, in which the 3D complexes serve as a model for cartilage tissue engineering. The alginate-chondrocytes 3D complexes were cultured for 21 days with or without Icariin treatment. Based on the above functional analysis, Icariin at the concentration of 10^−6^ M was selected for the 3D cultures. Histological analysis by H&E staining showed that most cells accumulated into clusters and appeared well-differentiated chondrocytes in Icariin treated group compared with the control group (Fig 6A). Alcian blue staining indicated that Icariin increased the production of proteoglycan including both sulfated and carboxylated acid mucopolysaccharides and sialomucins in the ECM of newly formed cartilage (Fig 6B and 6D). Many clustered chondrocytes were embedded in the metachromatic matrix (Fig 6B). SO staining also revealed a significant increase in acidic proteoglycan synthesis in the ECM of newly formed cartilage following Icariin treatment (Fig 6C). The total SO positive cartilage area in the sections from the 3D alginate-chondrocytes complexes treated with Icariin is almost 8 times more than the group without Icariin treatment (Fig 6E). These were accompanied by the upregulated mRNA expression of chondrogenic marker genes including *Sox9*, *Col2α1*, *Aggrecan* and the gene *Col10α1* that is only upregulated at the late stage of chondrogenic differentiation (Fig 6F–6I). Immunostaining confirmed the increased expression of the chondrogenic key transcription factor SOX9 at the protein level (Fig 6J) and the percentage of SOX9 positive chondrogenic cells in the Icariin treated group was around 2.3 times that of the control group (Fig 6K). The protein expression of chondrocyte marker collagen type 2 was also increased and the average integral optical density of the immunostaining in Icariin treated group was 2.4 times that of the control group (Fig 6J and 6L). Immunostaining for PCNA revealed that Icariin promoted chondrocytes proliferation in the alginate-chondrocyte 3D complexes as demonstrated by a significant increase in the percentage of PCNA positive chondrocytes (Fig 7A and 7B). These phenotypes were associated with the upregulation of HIF-1α protein expression and the significantly increased HIF-1α positive cell numbers (2 folds) in the 3D complexes treated with Icariin compared with the control group (Fig 7C and 7D). These results suggest that Icariin promotes chondrogenesis in the alginate-chondrocytes 3D cultures through promoting chondrocyte proliferation, differentiation and ECM synthesis in association with activation of HIF-1α in chondrocytes.

### Icariin promotes articular cartilage repair in the osteochondral defect model

**Fig 8:**
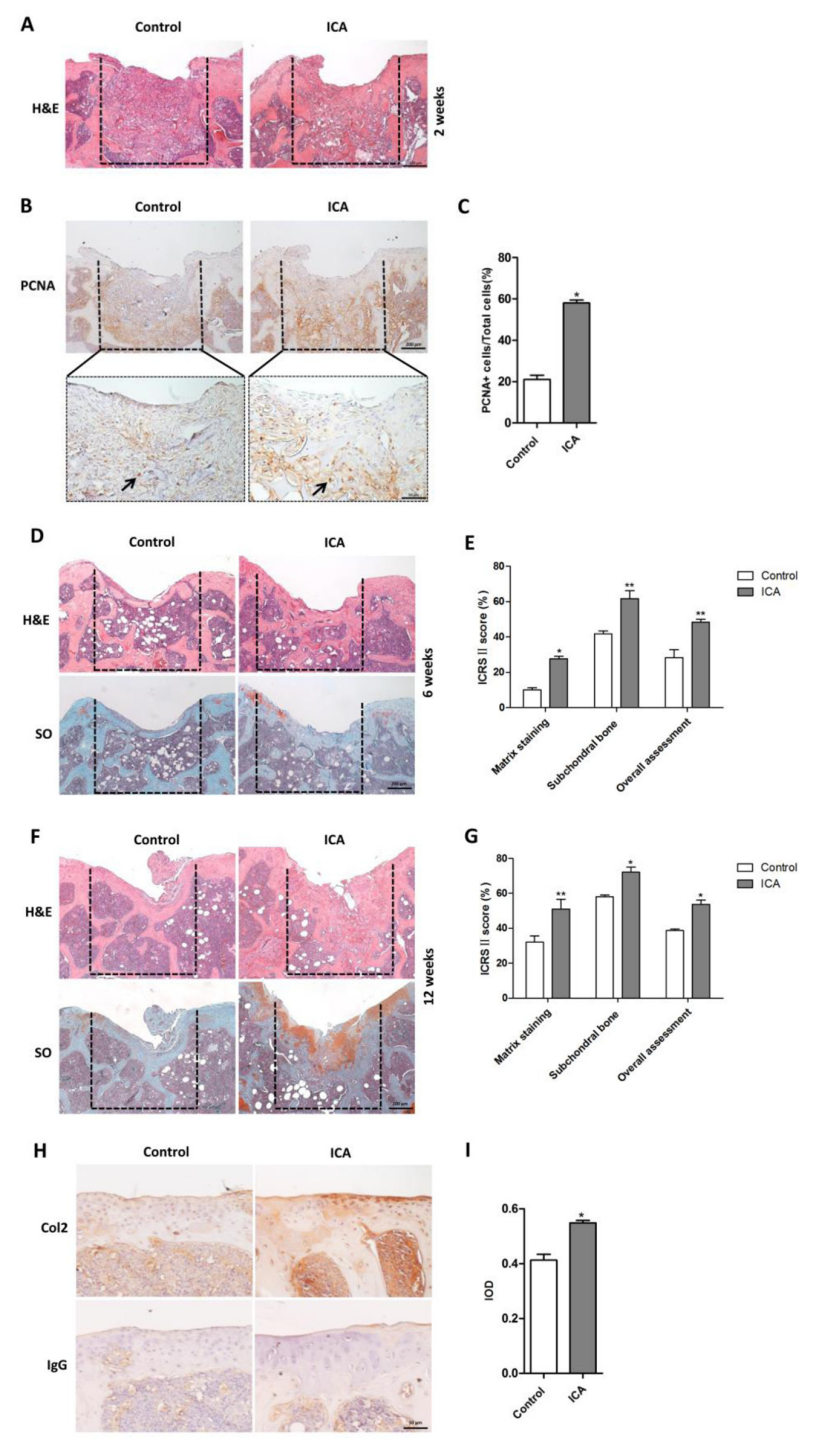
Icariin promotes articular cartilage repair in the mouse osteochondral defect model. 3D complexes incorporated with or without Icariin were transplanted in osteochondral defect regions of the distal femur of mice (detailed in Material and Methods). The dashed lines surround the newly formed tissue in the osteochondral defect region. (A) H&E staining showed the osteochondral defect regions in Icariin treated group and control group at 2 weeks post-transplantation. Scale bar = 200 μm. (B) Immunostaining for PCNA in the sections from icariin treated group and control group at 2 weeks post-transplantation. Upper panel, low magnification, Scale bar = 200 μm. Lower panel, high magnification, Scale bar = 50 μm. (C) Quantitation of the percentage of PCNA^+^ cells in the PCNA-stained sections represented in (B). *P < 0.05, n = 5. (D, F) H&E staining (upper panels) showed the osteochondral defect regions and SO staining (lower panels) indicated proteoglycan synthesis in the osteochondral defects in Icariin treated group and control group at 6 weeks (D) and 12 weeks (F) post-transplantation. Scale bar = 200 μm. (E, G) Quantitation of ICRS II cartilage repair score at 6 weeks (E) and 12 weeks (G) post-transplantation. The parameters shown include matrix staining (SO staining), subchondral bone and overall assessment. Icariin-treated group compared with control group, *P < 0.05, **P < 0.01, n = 5. (H) Immunostaining for Col2 in the newly formed cartilage tissue in Icariin treated group and control group at 12 weeks post-transplantation. IgG was used as negative control. Scale bar = 50 μm. (I) Densitometric analysis of Col2 immunostaining in (H) using GraphPad Prism 5 software. *P < 0.05, n = 5.

To further define the role of Icariin in articular cartilage repair *in vivo*, we constructed the 3D alginate-Gelfoam delivery system incorporated with or without Icariin. The alginate 3D complexes were transplanted into the osteochondral defect model in the inter-chondyle notch of the distal femur of the mice. At 2 weeks post-surgery, H&E staining showed that the cartilage defect regions were filled with amorphous mesenchymal tissues in both groups with no typical architecture of articular cartilage (Fig 8A). However, quantitation analysis revealed an increase in PCNA immuno-reactive cell numbers in the repair region in the group transplanted with Icariin-incorporated 3D alginate-Gelfoam complexes compared with the control group (Fig 8B and 8C). This was in accord with the positive effect of Icariin on proliferation of chondrocytes in the *in vitro* 3D culture system (Fig 7A and 7B). By 6 weeks, the osteochondral defects were filled with newly formed cancellous bone covered by a superficial layer of hyaline cartilage or fibrocartilage tissue in the group treated with 3D alginate-Gelfoam complexes incorporated with Icariin, while only fibrous connective tissues covering the cancellous bone were observed in the control group as indicated by H&E and SO staining (Fig 8D). ICRS II histological scoring showed that several selected parameters including matrix staining (SO+ stained area), subchondral bone and overall assessment were significantly higher in scores in the repair region in the group treated with 3D alginate-Gelfoam complexes incorporated with Icariin than that of the control group (Fig 8E). By 12 weeks, higher amounts of hyaline cartilage were observed to cover on the subchondral bone in the group treated with 3D alginate-Gelfoam complexes incorporated with Icariin as compared with that of the control (Fig 8F). This was accompanied by improvement in ICRS II histological scoring of the above parameters (Fig 8G). The newly formed cartilage showed more abundant expression of cartilage matrix Col2 in Icariin treated group than that of the control group (Fig 8H and 8I). These results suggest that Icariin facilitates the repair of articular cartilage defects by enhanced subchondral bone formation and restoration of hyaline cartilage coverage.

## Discussion

Being absent of vasculature, articular cartilage is formed and maintained in a hypoxic environment during development and repair [28–30]. Chondrogenic lineage cells including MSCs/chondroprogenitors and chondrocytes are readily located to sense the low oxygen tension and develop specific mechanisms to regulate proliferation, differentiation and ECM synthesis. Recent studies highlight the importance of the HIF-α pathway in the development, homeostasis or repair of cartilage in both humans and animals [14–16, 31–36]. Our recent data show that prolyl hydroxylase inhibitors activate HIF-1α and enhances skeletal repair through promoting angiogenesis and cartilaginous callus formation [20]. Local delivery of EPO, an HIF-α downstream target, promotes bone healing through enhancing cartilaginous callus formation and angiogenesis [21]. The information indicates that manipulation of the HIF-α pathway may develop novel therapeutic approach for articular cartilage repair. In the present study, we provide the evidence that HIF-1α in the chondrogenic cells can be pharmacologically targeted using flavonoid compound Icariin to enhance chondrogenesis in micromass and 3D alginate hydrogel culture systems and to promote articular cartilage repair in a mouse osteochondral defect model.

Icariin is an active flavonoid glucoside isolated from Herba Epimedii registered in the Chinese pharmacopoeia. Previous studies indicate that Icariin possesses wide pharmacological effects including cardiovascular protection [37], neuron protection [38], osteogenic promotion [39–41], bone resorption inhibition [42,43], anti-inflammation [44,45] and tumor growth inhibition [46]. In recent years, Icariin has been found to improve the restoration of supercritical-sized osteochondral defects in rabbit [47]. The effect is at least partly attributed to the ability of Icariin to promote chondrogenic gene expression and ECM synthesis that were observed in rabbit or murine chondrocytes and rat MSCs [47–50]. However the underlying mechanisms and the targeted signaling pathways remain unknown.

In an attempt to screen and identify natural compound small molecules that have the ability to activate the HIF-1α pathway, we found that Icariin had a positive effect on the hypoxia response element activity. Chondrocytes treated with Icariin showed accumulation of HIF-1α with increased nuclear localization compared with the controls. The upregulation of *HIF-1α* persisted during chondrogenic differentiation for 7 and 14 days upon Icariin treatment. Our experiment, in which the Icariin-induced HIF-1α accumulation in chondrocytes was eliminated by the addition of FeSO_4_ (the donor of iron ion (Fe^2+^)), suggests that the HIF-1α accumulation is likely a consequence of the inhibited PHD activity due to insufficient cellular iron ions, which work as a required cofactor for PHD to function but are deprived of by a potential interaction with Icariin when Icariin is present in the culture. This process shows a certain similarity to the upregulation of HIF-1α under hypoxia, where PHD activity is inhibited due to the deprivation of O2. And we observed a comparable HIF-1α accumulation in hypoxic chondrocytes and chondrocytes treated with Icariin, though an additive or synergistic effect was not observed in chondrocytes treated with both Icariin and hypoxia. Interestingly, we found that the protein expression of PHD2 and PHD3 in chondrocytes was also reduced by Icariin treatment. How Icariin regulates PHD expression and whether the reduction in PHD expression also contributes to the HIF-1α accumulation and/or has other biological significance in chondrocytes, need to be further studied.

We next performed safety and functional assays in chondrocytes *in vitro*. Different from the previous study in MC3T3-E1 preosteoblastic cell line, where Icariin (10^−10^ to 10^−5^ M) showed low degree of cytotoxicity with cell viability varied from 88% to 98% in a 3-days of culture [51], we did not observe obvious cytotoxicity in chondrocytes. Icariin at the concentration of 10^−6^ M significantly increased the proliferation of chondrocytes or chondroprogenitors indicated by MTT assay, BrdU incorporation and colony formation assays respectively, while its effects declined to the basal level when the concentration reached 10^−5^ M. This suggests that Icariin is a safe candidate molecule to treat chondrocytes and 10^−6^ M might be its optimal concentration to promote chondrocyte proliferation. The proliferation-promoting effect of Icariin on chondrocyte was further evidenced in the 3D alginate hydrogel culture system, in which the Icariin-treated complexes generated significantly higher PCNA-positive chondrocytes than untreated complexes. It has been reported that HIF-1α is a negative regulator of proliferation in chondrocytes [14] and some cancer cells [52]. Interestingly, we observed that the positive effect of Icariin on chondrocyte proliferation was eliminated when HIF-1α was deleted. This data together with the above information suggests that Icariin treatment appears to promote chondrocyte proliferation at least partially mediated by HIF-1α, while the HIF-1α-independent mechanism might also be involved which could not be resolved here. In addition to the proliferation-promoting effect, Icariin (10^−6^ M) increased the mRNA expression of chondrogenic genes (*Sox9*, *Col2α1* and *aggrecan*) and the production of cartilaginous matrix in both the micromass culture and the 3D culture system. Moreover, an enhanced chondrogenesis was observed in the 3D alginate hydrogel culture system treated with Icarrin (10^−6^ M), which was characterized by upregulation of late chondrocyte differentiation marker *Col10α1*, increased Col2 synthesis in the ECM and larger cartilage area. These observations are in accord to the recent studies in other species including rabbit [47,48], murine [49] and rat [50].

Notably we detected the upregulation of HIF-1α protein expression and significantly increased HIF-1α positive cell numbers in the 3D complexes treated with Icariin compared with that without Icariin treatment. Further analysis by using a Cre-mediated HIF-1α deletion model revealed that Icariin relied on HIF-1α to increase cartilaginous matrix synthesis. Deletion of HIF-1α in chondrocytes reversed the increased expression of anabolic marker genes in chondrocytes in response to Icariin treatment, suggesting a HIF-1α-dependent mechanism involved in the regulation of chondrocyte anabolic function by Icariin. Deletion of HIF-1α showed diverse effects on chondrocyte catabolic marker genes expression in response to Icariin treatment. The decreased mRNA expression of Adamts4, MMP2 and MMP9 upon Icariin treatment was eliminated, further enhanced and reduced respectively by HIF-1α deletion, which also indicates that the control of chondrocyte catabolic function by Icariin is HIF-1α-independent and the underlying mechanism seems more complicated which deserves to be further characterized. Thus, the promoting effect of Icarrin on anabolic functions together with its inhibiting effect on the transcription of key catabolic genes may contribute together to the enhanced chondrogenesis by improving the cartilage matrix generation, which is regulated by HIF-1α. HIF-1α was identified as a key regulator for chondrocyte differentiation through direct binding to the promoter region of Sox9 gene [15]. It deserves to note that contrary results exist regarding the relationship between Sox9 and HIF-1α expression in chondrogenic cells under hypoxia in different experimental settings [53, 54]. And HIF-1α is defined to play critical role in joint development [54]. In line with the increased HIF-1α^+^ cell numbers and elevated HIF-1α protein level in chondrocytes of the newly formed cartilage in the Icariin treated 3D complexes, we observed upregulated protein expression of the chondrogenic key transcription factor SOX9 as well as increased SOX9^+^ chondrogenic cell numbers, implying that an enhanced chondrocyte differentiation in association with activation of HIF-1α may underlie the Icariin-promoted chondrogenesis.

The chondrogenesis-promoting effect of Icariin highlights its potential use in cartilage repair or regeneration. Indeed, in the mouse osteochondral defect model, we found that alginate hydrogel 3D complexes incorporated with Icariin significantly enhanced articular cartilage repair compared with the non-drug controls indexed by improved ICRS II histological score of the repair region. It is interesting to note that Icariin increased SO+ cartilage area is accompanied by enhanced subchondral bone formation. This supports the notion that the integration of the superficial layer of cartilage and subchondral bone is a key element for functional articular cartilage repair or regeneration. Icariin may exerts positive effects on promoting the integration of the superficial layer of cartilage and subchondral bone, while the accurate control of this process by Icariin deserves to be further investigated. Taken together, our results suggest that Icariin may function as an HIF-1α activator to promote articular cartilage repair through coordinating chondrocytes proliferation, differentiation and integration with subchondral bone formation.

It is worth noting that there exists other HIF-α isoforms, HIF-2α and HIF-3α, which may also respond to Icariin but play different roles in regulation of chondrogenesis. For instance, HIF-1α is primarily involved in cartilage formation and maintenance while HIF-2α is crucial in articular cartilage homeostasis [55,56]. Of note, conditional mutagenesis reveals that HIF-2α does not seem to play major roles in the growth plate during endochondral ossification [57]. Because of the complexity of the potential interaction and distinct functions of HIF-α subunits, we could not rule out the potential impact of Icariin on HIF-2α or HIF-3α in chondrocytes, which deserves further investigation.
